# Observations on Acid Rain

**Published:** 1846-03

**Authors:** 


*Observations on Acid Rain.* By M. Ducres, (Journ. de Pharm., April, 1845 )—During the early part of June, 1842, a storm occurred in the town of Nismes, accompanied with much thunder and a large amount of hail. From some peculiarity in the taste of the hail, the author was led to examine it more closely. Having collected a quantity of it, and allowed it to melt, it was found to have an acid reaction, which, upon examination, was found to be due to nitric acid, formed no doubt by the action of electric fluid on the elements of the atmosphere. The occurrence of nitric acid in hail is not new, but the statement of this fact goes to confirm observations previously made.—*American Journal of Science and Arts.*

